# Comparative Transcriptomics and Methylomics Reveal Adaptive Responses of Digestive and Metabolic Genes to Dietary Shift in Giant and Red Pandas

**DOI:** 10.3390/genes13081446

**Published:** 2022-08-14

**Authors:** Lu Li, Fujun Shen, Xiaodie Jie, Liang Zhang, Guoqiang Yan, Honglin Wu, Yan Huang, Rong Hou, Bisong Yue, Xiuyue Zhang

**Affiliations:** 1 Key Laboratory of Bio-Resources and Eco-Environment, Ministry of Education, College of Life Sciences, Sichuan University, Chengdu 610065, China; 2 The Sichuan Key Laboratory for Conservation Biology of Endangered Wildlife, Chengdu Research Base of Giant Panda Breeding, Chengdu 610081, China; 3 Sichuan Key Laboratory of Conservation Biology on Endangered Wildlife, College of Life Sciences, Sichuan University, Chengdu 610065, China; 4 China Conservation and Research Center for the Giant Panda, Dujiangyan 611800, China

**Keywords:** giant panda, red panda, adaptive evolution, dietary shift, stomach, small intestine, convergent differentially expressed genes, methylation regulation

## Abstract

Both the giant panda (*Ailuropoda melanoleuca*) and red panda (*Ailurus fulgens*) belong to the order Carnivora, but have changed their dietary habits to eating bamboo exclusively. The convergent evolution characteristics of their morphology, genome and gut flora have been found in the two pandas. However, the research on the convergent adaptation of their digestion and metabolism to the bamboo diet, mediated by the dietary shift of the two pandas at the gene-expression and epigenetic regulation levels, is still lacking. We therefore used RNA sequencing among five species (two pandas and three non-herbivore mammals) and bisulfite sequencing among three species (two pandas and a carnivore ferret) to sequence key digestion and metabolism tissues (stomach and small intestine). Our results provide evidence that the convergent differentially expressed genes (related to carbohydrate utilization, bile secretion, Lys and Arg metabolism, vitamin B12 utilization and cyanide detoxification) of the two pandas are adaptive responses to the bamboo diet containing low lipids, low Lys and Arg, low vitamin B12 and high cyanide. We also profiled the genome-wide methylome maps of giant panda, red panda and ferret, and the results indicated that the promoter methylation of the two pandas may regulate digestive and metabolic genes to adapt to sudden environmental changes, and then, transmit genetic information to future generations to evolve into bamboo eaters. Taken together, our study provides new insights into the molecular mechanisms of the dietary shift and the adaptation to a strict bamboo diet in both pandas using comparative transcriptomics and methylomics.

## 1. Introduction

Convergent adaptive evolution in distant species can be affected by similar selective pressures of the environment [1,2]. The emergence of phenotypic convergence results from genetic convergence, such as convergent amino acid substitution, convergent gene recruitment, and the pathways of the same metabolism and regulation [3,4,5]. Diet plays a crucial role in driving the evolution of species, and thus, the convergent phenotype evolution of morphology, physiology and behavior is common across taxa with similar dietary habits [6,7,8].

The giant panda and red panda, as herbivores in the order Carnivora, are ideal models for studying convergent evolution driven by diet. To adapt to high-fiber and low-nutrition bamboo, convergent evolution has been found at multiple levels of the two pandas, for example: their extra pseudo-thumbs for the efficient grasping of bamboo [9,10]; similar skull traits to adapt to tough bamboo [11,12,13]; higher gene abundance involved in starch and sucrose metabolism in the gut microbiota for both pandas to meet their energy demands [14,15]; and the convergent evolution of serine protease genes (*PRSS1*, *PRSS36*, *CPB1*) for the release of Lys and Arg from the proteins of food and amino acid recycling to compensate for the deficiency in bamboo; and the adaptive convergence of two genes *GIF* and *CYP4F2* for the efficient absorption of vitamin B12 to alleviate the cardiovascular diseases caused by a vitamin B12-deficient diet [9]. Similarly, both pandas have a more functional bitter-taste receptor gene *TAS2R* than other carnivores to sense the bitterness of bamboo [16], and high proportions of Pseudomonadaceae in their gut microbes for cyanide detoxification [17] to response to the extraordinarily rich bitter and toxic cyanide compounds in bamboo [18]. These studies partly explain the adaptive evolution of the two pandas to specific dietary alteration at the morphology, genome and gut microbiota levels. However, gene expression and regulation are more flexible and critical in environmental adaptation [19,20,21], while the way in which the expression and regulation of genes related to digestion and metabolism in the two pandas respond to their unique dietary shift is still unclear.

Exploring the expression changes in the metabolism-related genes in both pandas compared with non-bamboo-eating omnivores and carnivores will help us to further understand the molecular mechanisms of adaptation to their bamboo diet. Therefore, we performed RNA sequencing (RNA-seq) of stomach and small intestine tissues from adult giant panda, red panda and ferret (*Mustela putorius furo*) using next-generation sequencing and carried out a comparative analysis using the RNA-seq data on stomach and small intestine tissues in mouse (*Mus musculus*) and dog (*Canis lupus familiaris*). In addition, DNA methylation, which can regulate gene expression without a sequence change, plays an important role in the adaptive evolution of species [22]. We also performed Bisulfite sequencing (BS-seq) of stomach and small intestine tissues from adult giant panda, red panda and ferret to further explore the epigenetic regulation mechanisms of metabolism-related differentially expressed genes (DEGs) in the two pandas.

## 2. Materials and Methods

### 2.1. Sample Collection

The tissue samples of adult giant panda and red panda were provided by the Chengdu Research Base of Giant Panda Breeding in Chengdu and the China Research and Conservation Center for the Giant Panda in Dujiangyan, Sichuan Province, China. The wild giant panda adults were seriously injured when found during ecological investigations and died during the rescue attempt. Except for one red panda, who died of lung cancer, all the red panda adults used in this study died of natural causes. tissue samples from healthy ferret adults were provided by Wuxi Kuboyi Pet Products Co., Ltd. (Wuxi, China). The ferret adults were anesthetized and euthanized, and their stomach and small intestine tissue samples were dissected in our laboratory.

Three stomach (aml_9775, aml_JV, aml_WE) and four small intestine (aml_9552, aml_PXC, aml_XC, aml_TC3) tissue samples were obtained from giant panda adults. Four stomach (afu_5808, afu_5809, afu_5815, afu_YMW) and four small intestine (afu_5812, afu_CC, afu_Z133, afu_YMC) tissue samples were obtained from red panda adults. Three stomach (mpf_W1, mpf_W2, mpf_W3) and three small intestine (mpf_XC1, mpf_XC2, mpf_XC3) tissue samples were obtained from ferret adults. There were no pathological changes in any of the stomach or small intestine tissue samples. All the samples were stored at −80 °C before RNA or DNA extraction.

Additional high-quality RNA-seq libraries of stomach and small intestine tissue samples from healthy adult animals (mouse and dog) were downloaded from the NCBI Short Read Archive (SRA) database.

### 2.2. Library Preparation and RNA Sequencing

The total RNAs from the stomach and small intestine tissue samples of giant panda, red panda and ferret for RNA-seq library construction were extracted using a TRIzol reagent (Invitrogen, Carlsbad, CA, USA) according to the manufacturer’s protocol, including treatment with DNase. The sequencing libraries were prepared using NEBNext^®^ UltraTM RNA Library Prep Kit for Illumina^®^ (New England Bio-Labs, Ipswich, MA, USA) following the manufacturer’s instructions. The index codes were added to attribute the sequences to each sample. After column purification, the quality of the resulting libraries was assessed using the Agilent Bioanalyzer 2100 system. The library preparations were sequenced using the Illumina HiSeq2000 platform in Novogene (Beijing, China) or Berry Genomics (Beijing, China).

### 2.3. RNA-Seq Read Mapping

The reference genomes and annotations of giant panda and red panda were obtained from NCBI ASM200744v2 and DNA ZOO (https://www.dnazoo.org/ accessed on 27 June 2022), respectively. The reference genomes and annotations for other species were obtained using Ensembl release 101 (Table S1). Low-quality reads and any adapter sequences were removed using fastp [23] with a quality score of 20. We mapped the high-quality reads that passed the filter thresholds to the animal genomes using HISAT2 [24]. Then, we converted the alignments from SAM to BAM format using SAMtools [25]. After reading in the reference annotations to count the fragments, a count of all the exons, grouped by gene, was calculated using featureCounts [26].

### 2.4. RNA-Seq Analysis

We identified 1:1 single-copy orthologous genes among the different species using Orthofinder 2.3.7 software [27]. The orthologous gene IDs and symbols of giant panda were used as proxies for the gene descriptions. The RNA-seq of cross-species comparison will result not only in different gene lengths but also in different sequencing depths, and the comparable expression levels of orthologous genes among the species can be obtained via the method combined gene-length correction with the trimmed mean of M-values (TMM) normalization procedure (applied to edgeR) [28]. We constructed gene expression matrices of stomach and small intestine samples separately, with each column presenting a sample and each row presenting the expression of an ortholog. Genes with low expression were filtered to include only genes expressed in counts greater than zero in the samples of the same species. Variance filtering can remove genes with fewer differential expressions and relatively less significance in the samples, alleviating high dimensionality and multiple testing issues [29,30]. We then proceeded to perform variance filtering with a threshold of 0.5. We defined each species as a group and a set of scaling factors was computed using TMM to normalize the library sizes. Normalized GeTMM values were used in the downstream analyses.

The normalized gene expression matrices of all samples were log-transformed. Principal component analysis (PCA) was performed on these converted data using the “prcomp” function in the R package “stats”. The Spearman correlation distance between samples was analyzed using the “cor” function; then, it was visualized using “heatmap.2” in the “gplots” package.

Expression analyses of DEGs on normalized GeTMM values were performed using the R “edgeR” package [31]. The giant panda and red panda samples were compared with other non-herbivore samples separately. DEGs shared between each panda and the other non-herbivorous species were identified as convergent DEGs. Significant DEGs were considered to be those with a Padj < 0.05 and an absolute value of log2 fold change > 1. Heat maps of convergent DEGs in the stomach and small intestine samples were drawn using the “heatmap” function in the R “pheatmap” package.

The expressed genes of the giant panda were selected as the background in the functional enrichment analysis. Gene ontology (GO) and Kyoto Encyclopedia of Genes and Genomes (KEGG) enrichment analyses were performed using the g:Profiler (http://biit.cs.ut.ee/gprofiler/gost accessed on 27 June 2022) online software and KOBAS (http://bioinfo.org/kobas/ accessed on 27 June 2022) online software, respectively.

### 2.5. Library Preparation and Bisulfite Sequencing

The stomach and small intestine samples of adult giant panda, red panda and ferret were used to perform whole-genome bisulfite sequencing (WGBS), exploring the potential epigenetic regulatory role of DNA methylation in the two pandas.

The genomic DNA of the tissues for WGBS was extracted using the Qiagen DNeasy Blood & Tissue Kit. An amount of 5.2 μg of genomic DNA spiked with 26 ng lambda DNA was used to prepare each Illumina library. At first, total DNA was sonicated to 200–300 bp using a Covaris S220, followed by end repair and adenylation. Then, cytosine methylation barcodes were ligated to sonicated DNA. The DNA fragments were bisulfite converted using the EZ DNA Methylation-GoldTM Kit. Finally, the DNA fragments were amplificated using the KAPA HiFi HotStart Uracil + ReadyMix (2X). The concentration of the resulting libraries was quantified using the Qubit^®^ 2.0 Flurometer. The library preparations were sequenced using the Illumina Hiseq X-10 platform in Novogene (Beijing, China) or Berry Genomics (Beijing, China), and 150 bp paired-end reads were generated.

### 2.6. BS-Seq Read Mapping and Data Processing

Low-quality reads and any adapter sequences were trimmed prior to mapping using Trim Galore with the parameters -q 20 -length 20 (https://www.bioinformatics.babraham.ac.uk/projects/trim_galore/ accessed on 27 June 2022). Then, the reads were aligned to the corresponding reference genome using Bismark_v0.20.0 [32] with the default minimum alignment score function and sorted using Samtools [25].

Duplicate reads were removed using the deduplicate_bismark script in Bismark_v0.20.0. The number of converted and unconverted cytosines covering each locus was extracted using the bismark_methylation_extractor script in Bismark_v0.20.0.

The methylation level of each C site was calculated according to the following formula: Methylation level of C site=mC/(mC+C). *mC* and *C* represent the number of methylated and unmethylated reads, respectively. We divided the sequence into 10 kb/bin and calculated the methylation level of the sequence. The methylation level of each bin was calculated according to the following formula: Methylation level of bin= ∑mC/ ∑(mC+C).

Gene regions with an upstream bp of 2000 and a downstream bp of 2000 were divided into 20 bins, and the average methylation levels of each bin were also detected. Gene functional regions were defined using GenomicFeatures [33]. The methylation levels of the different gene functional regions, including the promoter (upper 1000 bp of the transcript start sites), gene, exon and intron were calculated.

### 2.7. Promoter Methylation Analysis

DNA methylation at the promoter level was analyzed due to a negative correlation between promoter methylation and the gene expression level. The promoter methylation levels were calculated using the formula: Methylation level of promoter=∑mC/ ∑(mC+C). *mC* and *C* represent the number of methylated reads in the promoter and the number of unmethylated reads, respectively. We constructed promoter-methylation-level matrices of stomach and small intestine samples separately, with each column presenting a sample and each row presenting the promoter methylation level of an ortholog with variance filtering.

PCA of promoter methylation data was performed using the “prcomp” function in the R package “stats”. The Spearman correlation distances between the samples were analyzed using the “cor” function; then, they were visualized using “heatmap.2” in the “gplots” package.

The differences in the promoter methylation levels between the two groups were identified with Padj < 0.05 using the one-tailed Wilcoxon rank-sum test. Orthologous genes with differentially methylated promoter regions of giant panda and red panda samples were compared with ferret samples, respectively. Genes with differentially methylated promoter regions shared between each panda and ferret were identified as convergent promoters. The expressed genes of the giant panda were selected as the background in the functional enrichment analysis. GO and KEGG enrichment analyses of the convergent promoters were performed using the g:Profiler (http://biit.cs.ut.ee/gprofiler/gost accessed on 27 June 2022) online software and KOBAS (http://bioinfo.org/kobas/ accessed on 27 June 2022) online software, respectively.

## 3. Results

### 3.1. Transcriptomic PCA and Clustering Analyses

To investigate the molecular mechanisms of the adaptive response to the specific bamboo diet of the two pandas, RNA-seq libraries of nine stomach and nine small intestine samples in giant panda, red panda and ferret were constructed. We also downloaded RNA-seq raw data of dog and mouse stomach and small intestine tissues from NCBI for joint analyses. The descriptions of the 32 tissue samples of the five mammals for RNA-seq obtained from various sources are provided in Table S1. All the RNA-seq data generated 19 to 116 million clean paired-end reads. We aligned each of the 32 cDNA libraries to their corresponding reference genome and found that the final efficiency of the RNA-seq read alignments ranged from 79.52 to 98.7% (Table S1). Table S2 shows the statistics of the orthologous gene numbers detected using Orthofinder software, including the number among the five species, the number among the two pandas and the ferret, the number between each panda and each other non-herbivore, and the number of the two pandas. We identified 10,121 orthologous genes among the five species. The protein sequence coverage and identity of the 10,121 orthologous genes among the five species are shown in Tables S3 and S4, respectively. The CV (ratio of the standard deviation to the mean) of data in the stomach and small intestine samples was lower after normalization, suggesting a feasible method to correct bias among species and individuals (Figure S1).

**Figure 1:**
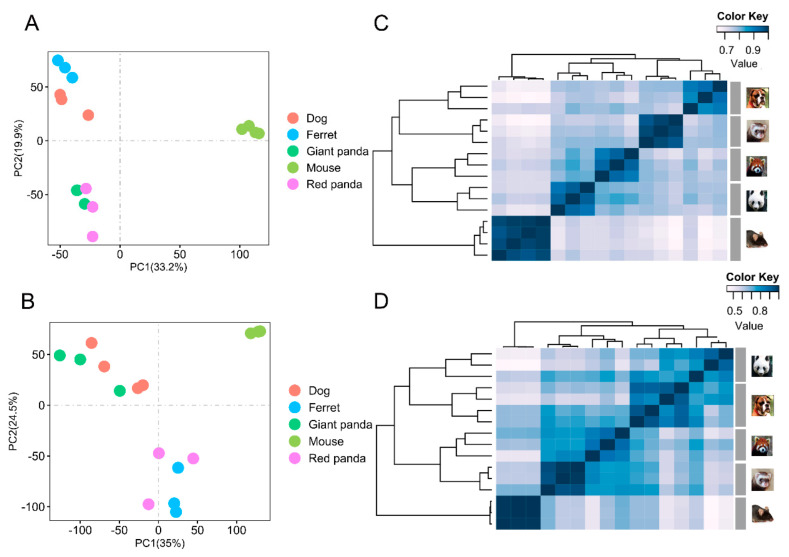
PCA and clustering analyses of the mRNA expressions for all samples. The expression levels of all orthologous genes were normalized and log-transformed to perform PCA in stomach samples of all species. Different species are represented by circles of different colors (**A**). The expression levels of all orthologous genes were normalized and log-transformed to perform PCA in small intestine samples of all species. Different species are represented by circles of different colors (**B**). The expression levels of all orthologous genes were normalized and log-transformed to perform cluster analysis in stomach samples of all species. Distance between samples was measured using Spearman’s rank correlation coefficient (**C**). The expression levels of all orthologous genes were normalized and log-transformed to perform cluster analysis in small intestine samples of all species. Distance between samples was measured using Spearman’s rank correlation coefficient (**D**).

PCA across species showed that the biological duplications of the same tissues clustered well (Figure 1A,B). The gene expression patterns were investigated using hierarchical clustering analyses based on Spearman’s correlation coefficients from the stomach and small intestine tissues of all the species. In the stomach, three major branches were identified: one representing dog and ferret, one representing giant panda and red panda, and the other representing mouse (Figure 1C). In the small intestine, three major branches were also identified: one representing giant panda and dog, one representing red panda and ferret, and the other representing mouse (Figure 1D). The difference in clustering patterns between the stomach and small intestine may be due to distinct response signatures with the tissue specificity of the gene expressions [34,35]. The overall small intestine gene expression patterns of the two pandas may be relatively more conservative.

### 3.2. Transcriptomic Profiles of the Stomach

**Figure 2:**
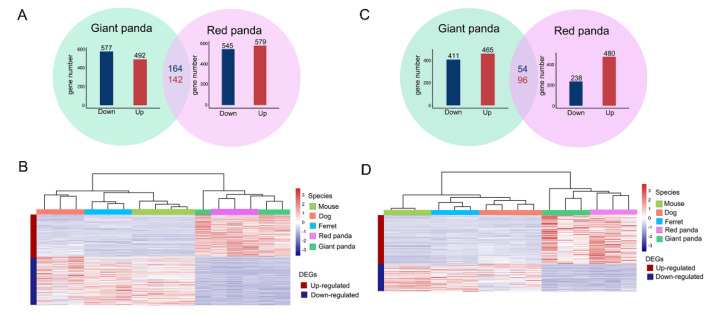
Transcriptional patterns in stomach and small intestine samples. Bar plots in green and purple circles indicate the numbers of DEGs in stomach samples of giant panda and red panda compared with all non-herbivore species, respectively. Numbers in red and blue indicate convergent up- and down-regulated DEGs of two pandas compared with all non-herbivore species in stomach samples, respectively (**A**). The expression levels of convergent DEGs of stomach samples were normalized and log-transformed to draw heat map by adopting hierarchical clustering method (**B**). Bar plots in green and purple circles indicate the numbers of DEGs in small intestine samples of giant panda and red panda compared with all non-herbivore species, respectively. Numbers in red and blue indicate convergent up- and down-regulated DEGs of two pandas compared with all non-herbivore species in small intestine samples, respectively (**C**). The expression levels of convergent DEGs of small intestine samples were normalized and log-transformed to draw heat map by adopting hierarchical clustering method (**D**).

We compared the stomach tissue samples of each of the panda species with each of the three non-herbivore species to obtain the DEGs, as shown by the volcano plots (Figure S2A). After taking the intersection, we obtained the stomach DEGs of each of the panda species compared with all the non-herbivore species. We found 492 up-regulated DEGs and 577 down-regulated DEGs in the stomach samples of giant panda compared with all the non-herbivore species. Similarly, we identified 579 up-regulated DEGs and 545 down-regulated DEGs in the stomach samples of red panda compared with all the non-herbivore species. These DEGs shared by the two panda species were identified as convergent stomach DEGs. Thus, we eventually found 306 convergent DEGs from the stomach samples of the two pandas, comprised of 142 up-regulated and 164 down-regulated convergent DEGs (Figure 2A and Table S5). In addition, we performed hierarchical clustering of these 306 convergent DEGs from the stomach samples. Two major branches were identified: one representing the panda group, and the other representing the non-herbivore group. The clustering results indicated that there were clear differences in the DEG expression patterns between the two bamboo-eating species and the three non-herbivore species (Figure 2B).

### 3.3. Transcriptomic Profiles of the Small Intestine

Consistent with comparisons of the stomach samples, we compared the small intestine tissue samples of each of the panda species with each of the three non-herbivore species to obtain the DEGs, as shown by the volcano plots (Figure S2B). After taking the intersection, we obtained the small intestine DEGs of each of the panda species compared with all the non-herbivore species. We identified 465 up-regulated DEGs and 411 down-regulated DEGs in the small intestine samples of giant panda compared with all the non-herbivore species. Similarly, we identified 480 up-regulated DEGs and 238 down-regulated DEGs in the small intestine samples of red panda compared with all the non-herbivore species. These DEGs, shared by the two panda species, were identified as convergent small intestine DEGs. Thus, we eventually identified 150 convergent small intestine DEGs in the two pandas, encompassing 96 up-regulated and 54 down-regulated convergent DEGs (Figure 2C and Table S6). The number of convergent DEGs identified in the small intestine samples was less than the number in the stomach samples. In addition, we performed hierarchical clustering of the 150 small intestine convergent DEGs. As for the small intestine samples, the clustering results indicated that there were also clear differences in DEG expression patterns between the two bamboo-eating panda species and the three non-herbivore species (Figure 2D).

### 3.4. Enrichment Analysis of Convergent DEGs and Identification of Digestion- and Metabolism-Related Convergent DEGs in the Stomach

**Figure 3:**
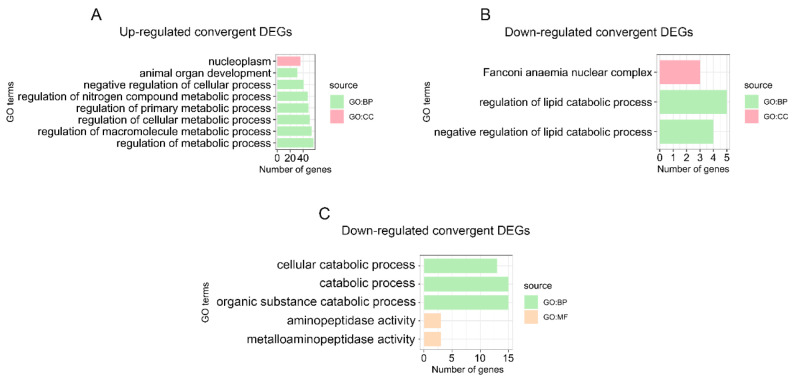
Significantly enriched GO categories for convergent DEGs. Up-regulated (**A**) and down-regulated (**B**) convergent DEGs of the two pandas in the stomach. Down-regulated (**C**) convergent DEGs of the two pandas in the small intestine. BP represents biological process, CC represents cellular component, and MF represents molecular function.

**Figure 4:**
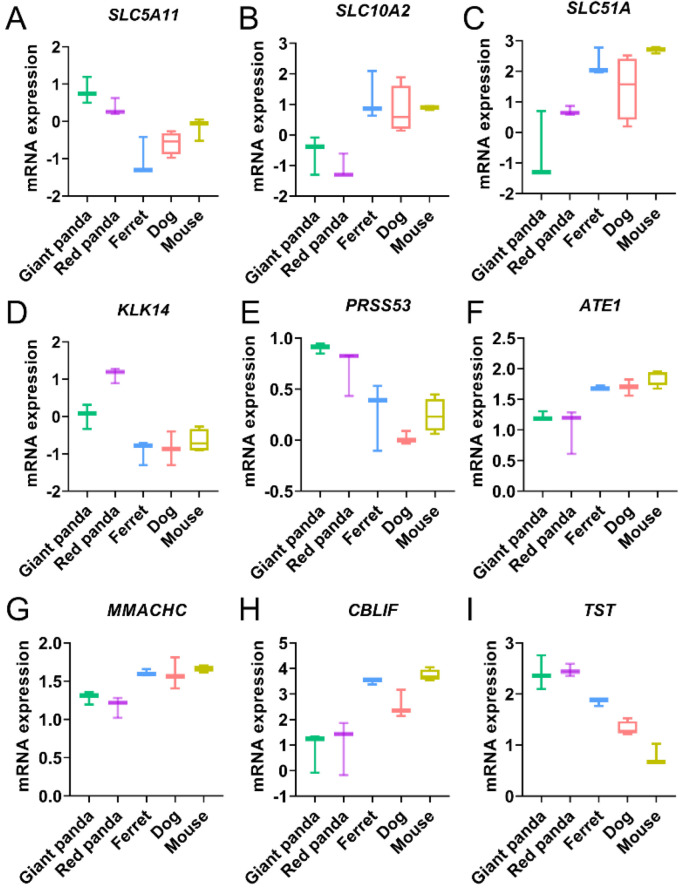
The expression trends of the convergent DEGs associated with carbohydrate utilization (**A**), bile secretion (**B**,**C**), Arg and Lys metabolism (**D**–**F**), vitamin B12 utilization (**G**,**H**) and cyanide detoxification (**I**) in stomach and small intestine samples. Y-axis represents normalized and log-transformed expression levels. Boxplot edges indicate the 25th and 75th percentiles. Different species are represented by different colors.

GO and KEGG analyses were performed to further understand the biological functions of the convergent stomach DEGs of the two pandas compared with all the non-herbivore species. The categories and pathways of the enrichment results are shown in Tables S7 and S8. The up-regulated convergent stomach DEGs of the two pandas were enriched to eight GO categories and no KEGG pathways. The up-regulated convergent DEGs of the two pandas were mainly involved in metabolic processes (Figure 3A and Table S7), such as the regulation of metabolic process (GO:0019222), the regulation of macromolecule metabolic process (GO:0060255), the regulation of cellular metabolic process (GO:0031323) and the regulation of primary metabolic process (GO:0080090). We identified three GO categories and no KEGG pathways, including the negative regulation of lipid catabolic process (GO:0050995) and the regulation of the lipid catabolic process (GO:0050994) in the down-regulated convergent stomach DEGs of the two pandas (Figure 3B and Table S8). We identified the up-regulated convergent DEGs (*SLC5A11*) related to carbohydrate utilization (Figure 4A and Table S5) in the stomachs of the two pandas. We found that the expression levels of the convergent stomach DEGs (*KLK14*, *PRSS53*), such as serine proteases related to Arg and Lys metabolism, were up-regulated (Figure 4D,E and Table S5). In addition, the expression levels of the convergent stomach DEGs (*MMACHC*, *CBLIF*) related to vitamin B12 utilization (Figure 4G,H and Table S5) were down-regulated in the two pandas.

### 3.5. Enrichment Analysis of Convergent DEGs and Identification of Digestion- and Metabolism-Related Convergent DEGs in the Small Intestine

The enrichment results of the GO categories and KEGG pathways for the convergent small intestine DEGs of the two pandas compared with all the non-herbivore species are shown in Tables S9 and S10. We identified five GO categories and one KEGG pathway, including the catabolic process (GO:0009056) and bile secretion (PATH: aml04976), in down-regulated convergent small intestine DEGs (Figure 3C and Tables S9 and S10). Meanwhile, the expressions of many convergent small intestine DEGs involved in bile acid transportation and bile secretion (*SLC10A2*, *SLC51A*) were down-regulated (Figure 4B,C and Table S6). We found that the expression of convergent small intestine DEG *ATE1* related to Arg metabolism was down-regulated (Figure 4F and Table S6). Moreover, we observed the up-regulated expression of convergent small intestine DEG *TST* related to cyanide detoxification (Figure 4I and Table S6).

### 3.6. DNA Methylation Patterns in Genomes

**Figure 5:**
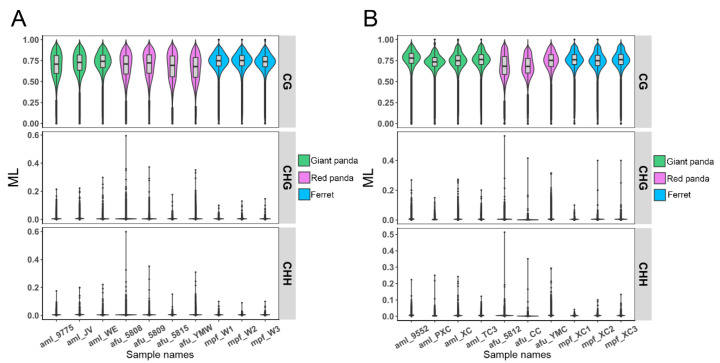
The distributions of methylation levels for the stomach (**A**) and small intestine (**B**) samples across the genomes of giant panda, red panda and ferret. The x-axis and y-axis represent different samples and methylation levels, respectively. Every 10 kb was considered a bin. The width of each violin represents the number of points below this methylation level.

To analyze the genome-wide methylation levels and the methylation regulation mechanism of nutrient metabolism in the two pandas, BS-seq libraries of ten stomach and ten small intestine samples in giant panda, red panda and ferret were generated. The description of the 20 tissue samples of three mammals for BS-seq is provided in Table S11. For each sample, a 100–138 G clean base was generated after data quality control (QC) performed, and the mapping efficiency ranged from 63.3 to 77.6% (Table S11). In addition, each accumulated distribution of sequence coverage at the corresponding genome is shown in Figure S3. Methylations in giant panda, red panda and ferret exist in three sequence contexts, including CG, CHG and CHH (H means A, T or C). More than 92.76%, 93.79% and 92.64% of the cytosine sites covered by at least one unique read were found in samples of giant panda, red panda and ferret, respectively. More than 75.69%, 64.61% and 57.02% of the cytosine sites covered by at least five unique reads were also found in the samples of giant panda, red panda and ferret (Table S12). The number and the methylation levels of the cytosines in the CG, CHG and CHH sites were calculated in each sample of giant panda, red panda and ferret (Figure S4). Whether in the stomach (Figure S4A,C), or small intestine (Figure S4B,D) samples of giant panda, red panda and ferret, the number of CG contexts covered by at least five reads was the least among the three contexts, whereas the number of CG contexts covered by at least five reads was the largest at methylation levels greater than 50%. Meanwhile, the overall distribution of methylation levels in the stomach (Figure 5A) and small intestine (Figure 5B) samples of giant panda, red panda and ferret are shown in the violin plots. The CG types exhibit high methylation levels with wide sections compared with the CHG and CHH types in both tissues of giant panda, red panda and ferret. These results suggest that DNA methylation occurred primarily in the CpG dinucleotide [36].

**Figure 6:**
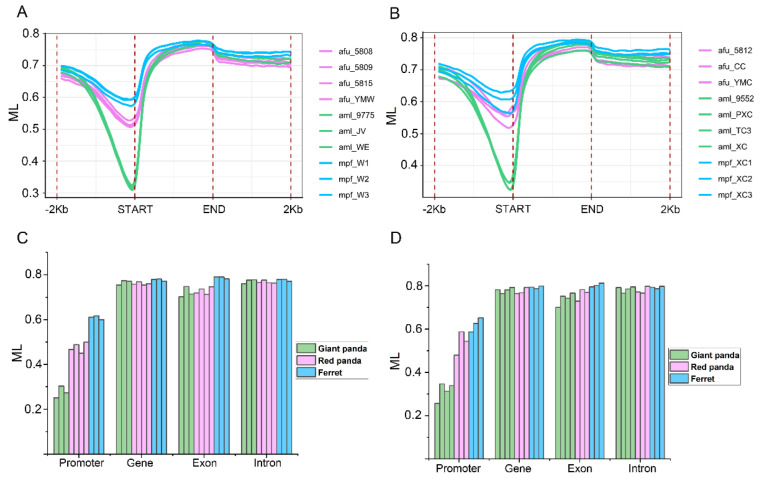
The DNA methylation patterns and levels. The methylation pattern diagram of gene regions with upstream 2000 bp and downstream 2000 bp regions in the stomach (**A**). The methylation pattern diagram of gene regions with upstream 2000 bp and downstream 2000 bp regions in the small intestine (**B**). The mean CG methylation levels in different gene elements including promoter, gene, exon and intron in the stomach (**C**). The mean CG methylation levels in different gene elements including promoter, gene, exon and intron in the small intestine (**D**).

CG methylation levels on a genome-wide scale, including the upstream, gene-body and downstream in the stomach (Figure 6A) and small intestine (Figure 6B) of giant panda, red panda and ferret, were calculated. Although CG methylation levels may not be very accurate due to a lack of region adjustment for overlapping genes, our results showed that the methylation patterns of giant panda, red panda and ferret were similar to those of other species [37,38,39,40]. In both tissues of three species, the methylation levels of 2000 bp upstream of the gene start site gradually decreased to the regions near the gene start site, which exhibited the lowest methylation levels in all of the regions. In general, in these regions with the lowest methylation levels of the two tissues, the methylation levels of red panda were much higher here than those of giant panda, and slightly lower than those of ferret. Subsequently, the methylation levels gradually increased to the regions near the gene end sites, and later, slightly decreased again. The methylated cytosines in the different gene elements, including the promoters, genes, exons and introns, have different functions [41]. Therefore, the average CG methylation levels were assessed in the stomach (Figure 6C) and small intestine (Figure 6D) samples of giant panda, red panda and ferret. Compared with other three gene elements, the lowest methylation levels in the promoter regions were found in the stomach (Figure 6C) and small intestine (Figure 6D) samples of giant panda, red panda and ferret. The promoter methylation levels of the giant panda samples were lowest among the three species in the stomach (Figure 6C) and small intestine (Figure 6D).

### 3.7. Promoter Methylation Profiles

We analyzed the promoter methylation of 10,984 orthologous genes detected using Orthofinder software in the stomach and small intestine of giant panda, red panda and ferret. Principal component analyses based on the promoter methylation-level data showed that the biological duplications of the stomach (Figure S5A) and small intestine (Figure S5B) samples in giant panda, red panda and ferret clustered well, while the samples of the different species separated. Hierarchical clustering analyses based on the promoter methylation-level data indicated that two major branches were identified in the stomach (Figure S5C) and small intestine (Figure S5D) according to the evolutionary relationship: one representing red panda and ferret, and one representing giant panda. 

We obtained the genes with differentially methylated promoter regions in the stomach of each panda compared with ferret. We identified 3177 hypomethylated and 711 hypermethylated promoters in the stomach samples of giant panda when compared with ferret. Similarly, when compared with ferret, we found 1829 hypomethylated and 1168 hypermethylated promoters in the red panda stomach. These genes with differentially methylated regions shared by the two pandas were identified as convergent promoters of the stomach. We found 1641 convergent promoters from the stomach samples of the two pandas when compared with ferret, comprised of 1210 hypomethylated and 431 hypermethylated convergent promoters (Figure S6A).

We obtained the genes with differentially methylated promoter regions in the small intestine of each panda compared with ferret. We identified 2696 hypomethylated and 585 hypermethylated promoters in the small intestine samples of giant panda when compared with ferret. Similarly, when compared with ferret, we found 1344 hypomethylated and 1055 hypermethylated promoters in the small intestine of red panda. These genes with differentially methylated regions shared by the two pandas were identified as convergent promoters of the small intestine. We found 1286 convergent promoters from the small intestine samples of the two pandas when compared with ferret, comprised of 908 hypomethylated and 378 hypermethylated convergent promoters (Figure S6B).

### 3.8. Methylation Levels of Convergent Promoters 

**Figure 7:**
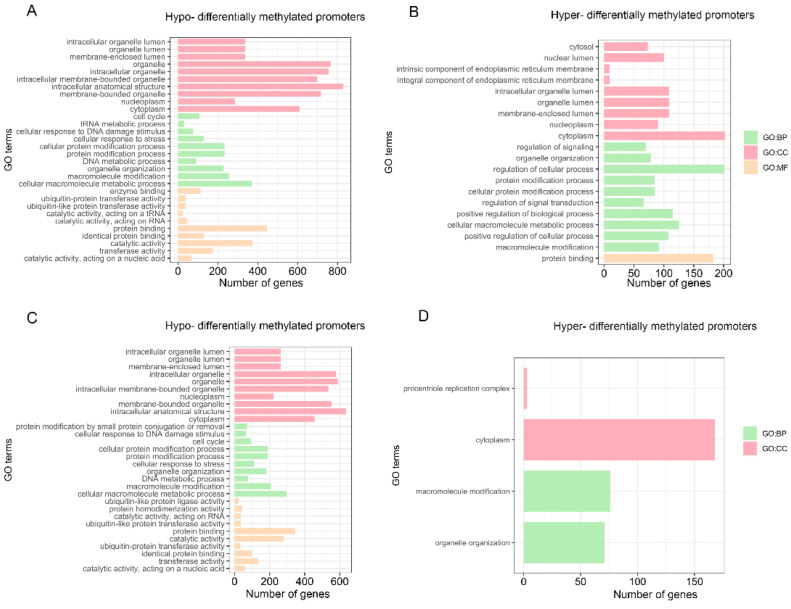
Significantly enriched GO categories for convergent promoters. Hypomethylated (**A**) and hypermethylated (**B**) convergent promoters of the two pandas in the stomach. Hypomethylated (**C**) and hypermethylated (**D**) convergent promoters of the two pandas in the small intestine. All Biological-process (BP), cellular-component (CC) and molecular-function (MF) categories with the top 10 most significantly enriched categories are displayed.

The enrichment of GO and KEGG analyses was performed on the convergent promoters of the two pandas, which are hypomethylated and hypermethylated compared with ferret in the stomach (Tables S13–S15). The hypomethylated convergent promoters of the two pandas were enriched to 194 GO categories and 0 KEGG pathways. Compared with ferret, the enrichment of the hypomethylated convergent promoters (Figure 7A and Table S13) includes the regulation of the metabolic process (GO:0019222), the protein metabolic process (GO:0019538), the regulation of the catabolic process (GO:0009894) and the metabolic process (GO:0008152). The hypermethylated convergent promoters of the two pandas were enriched to 35 GO categories and 1 KEGG pathway. Compared with ferret, the enrichment of the hypermethylated convergent promoters (Figure 7B and Tables S14 and S15) include the regulation of the protein metabolic process (GO:0051246), the regulation of the metabolic process (GO:0019222) and the protein metabolic process (GO:0019538).

Enrichment of the GO and KEGG analyses was also performed on the convergent promoters of the two pandas, which are hypomethylated and hypermethylated compared with ferret in the small intestine (Tables S16 and S17). The hypomethylated convergent promoters of the two pandas were enriched to 171 GO categories and 0 KEGG pathways. Compared with ferret, the enrichment of hypomethylated convergent promoters (Figure 7C and Table S16) includes the protein metabolic process (GO:0019538), the protein catabolic process (GO:0030163) and the regulation of the metabolic process (GO:0019222). The hypermethylated convergent promoters of the two pandas were enriched to four GO categories and no KEGG pathways. Compared with ferret, the enrichment of the hypermethylated convergent promoters (Figure 7D and Table S17) includes organelle organization (GO:0006996) and macromolecule modification (GO:0043412).

**Figure 8:**
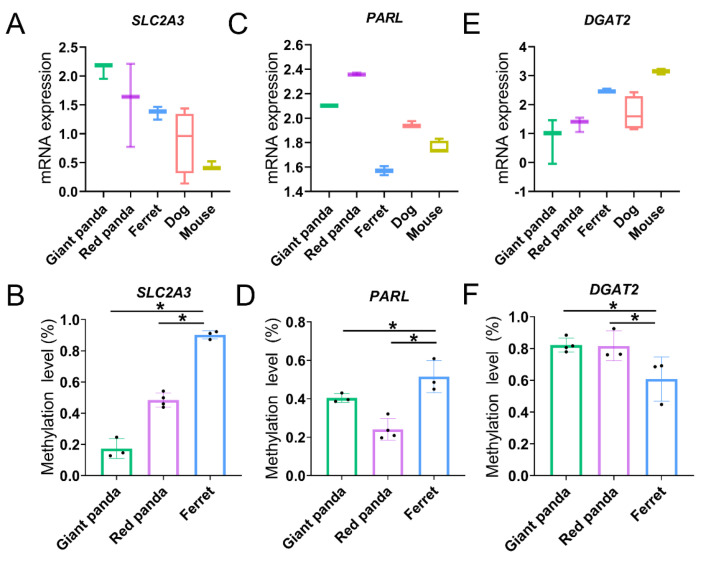
The mRNA expression (**A**,**C**,**E**) and promoter methylation (**B**,**D**,**F**) levels of DEGs in stomach and small intestine samples. * indicates Padj < 0.05.

Gene expression is usually negatively regulated by DNA methylation acting on the promoter region, and we compared each panda species with ferret to further focus on the methylation levels of the convergent promoters combined with the mRNA expression levels in the stomach and small intestine. In the stomach, compared with ferret, the convergent promoters in the two pandas of higher carbohydrate-utilization-related DEG *SLC2A3* (Figure 8A) were both hypomethylated (Figure 8B). Similarly, compared with ferret, the convergent promoters in the two pandas of higher Arg- and Lys-related stomach DEG *PARL* (Figure 8C) were also both hypomethylated (Figure 8D). In the small intestine, compared with ferret, the convergent promoters in the two pandas of lower lipid-absorption-related DEG *DGAT2* (Figure 8E) were both hypermethylated (Figure 8F).

## 4. Discussion

The giant panda and red panda are in different families within the order Carnivora, but both have evolved into bamboo-specialized herbivores. The stomach and the small intestine tissues play important roles in digestion and absorption, respectively. Our study is the first to describe the expression profiles of the convergent DEGs and convergent promoters in both pandas related to nutritious metabolism in the stomach and the small intestine using comparative transcriptomics combined with promoter methylation levels.

### 4.1. Convergent DEG Related to Carbohydrate Utilization

The giant panda and red panda need to effectively meet their daily energy requirements from bamboo, which has minimal lipid content [42,43]. Although protein is the material basis of all life, the cost of converting it into glucose to provide energy for life activities is very high. Carbohydrate, an energy-rich nutrient, is a valuable calorie resource from bamboo for the two pandas. A variety of carbohydrates in bamboo, including starches and sugars, are easily digested and absorbed [15]. The convergent stomach DEG *SLC5A11*, involved in the utilization of carbohydrates, was up-regulated in both panda stomachs (Table S18 and Figure 4A). SLC5A11, which can regulate the transport of glucose, may play a role in glucose-regulated feeding [44]. The high expression level of *SLC5A11* may help the two pandas meet the energy demands for their daily activities, growth, reproduction and metabolism by utilizing the highly bioavailable carbohydrates of low-nutrition bamboo. 

### 4.2. Convergent DEGs Related to Bile Secretion 

Dietary fats, mainly triglycerides, are predigested in the stomach by gastric lipase; then, they are further digested into smaller fragments in the small intestine by bile salts [45,46]. Two down-regulated convergent small intestine DEGs (*SLC10A2*, *SLC51A*) involved in bile secretion were found in the two pandas (Table S18 and Figure 4B,C). Bile acids are required for the emulsification and intestinal absorption of dietary lipids. Subsequently, most bile acids are reabsorbed from the intestinal lumen via the sodium/bile acid cotransporter SLC10A2 into the small intestinal cells and flow across the basolateral membrane via the intestinal basolateral transporter SLC51A into the portal blood [47,48,49]. 

The effective digestion and absorption of fats with very low aqueous solubilities is inseparable from the participation of bile acids [50]. The digested fats are absorbed by enterocytes, esterified to triglycerides, and then, enter into lipid metabolism [51]. Low dietary-fat intake may result in decreased lipid oxidation for the energy supply of the two pandas compared with non-herbivore animals. Meanwhile, carbohydrates are largely used for oxidation to meet the daily needs of the two pandas. The down-regulated expressions of genes related to bile secretion implies that both pandas are intolerant to a high-fat diet and adaptive to low-lipid bamboo diet compared with non-herbivore species.

### 4.3. Convergent DEGs Related to Amino Acid Metabolism

Compared with animal meats and plant leaves, much lower Lys and Arg content in bamboo was measured [9]. The lack of the essential amino acid Lys can lead to anemia and energy-metabolism disorders [52,53]. Arg can improve microvascular function, which has a complex impact on platelets, coagulation and fibrin solubility systems [54,55]. Two serine protease genes (*KLK14* and *PRSS53*) showed convergent up-regulation in the stomachs of the two pandas, which can release the Lys or Arg residue from the carboxy terminal protein (Table S18 and Figure 4D,E). The degradation of some proteins requires Arg mediated by arginyltransferase ATE1 [56]. Our study also found that the convergent DEG *ATE1*, related to Arg metabolism, was down-regulated in the small intestine (Table S18 and Figure 4F). These convergent DEGs of the two pandas may be an adaptive response to the bamboo diet with limited Lys and Arg content. 

### 4.4. Convergent DEGs Related to Vitamin Utilization and Cyanide Detoxification

For vegetarians, it is difficult to obtain vitamin B12, which can only be supplied by animal foods unless gut microbes are utilized to synthesize it [14,57,58,59]. A recent study suggested that MMACHC may play an important role in the conversion of vitamin B12 by processing and targeting it to destination enzymes [60]. CBLIF (cobalamin-binding intrinsic factor), a member of the cobalamin (vitamin B12) transport protein family, this glycoprotein secreted by the parietal cells of the gastric mucosa may promote the effective absorption of vitamin B12 in the small intestine. Accordingly, the adaptive convergence of stomach DEGs (*MMACHC*, *CBLIF*) may improve the deficiency in the nutritional supplement of vitamin B12 with the bamboo diet in the two pandas (Table S18 and Figure 4G,H).

Bamboo contains abundant toxic cyanide. The adult giant panda exhibits higher expression and activity of rhodanese, which plays a vital role in cyanide detoxification compared with domestic cat, and it is speculated that red panda may also undergo a similar rhodanese adaptation [61]. Typical carnivorous gastrointestinal systems, with short digestive tracts, brief digestion times and fast intestinal peristalsis, may result in higher oxygen concentrations, thus facilitating the growth of aerobic bacteria, such as the Pseudomonadaceae. Pseudomonadaceae may undergo convergent adaptation in the gut microbes of the two pandas, which is conducive to cyanide detoxification [17,18]. Thiosulfate Sulfurtransferase TST may convert toxic cyanide into thiocyanate to exert the effect of detoxification [62,63]. Up-regulated convergent DEG *TST* in the small intestine of the two pandas (Table S18 and Figure 4I) may play a role in accelerating the cyanide detoxification of bamboo.

### 4.5. DNA Methylation Regulation

Species-specific phenotypic traits are also due to differences in the regulation of gene expression levels across species. Epigenetic modification has great importance in gene expression, the preservation of genome integrity, phenotypic plasticity and adaptive evolution [64]. DNA methylation, an essential epigenetic modification involved in the regulation of numerous biological processes, may contribute to phenotypic variability, species-specific adaptation and species evolution [22,65]. A high density of CpG dinucleotides is necessary to induce a methylation-free state [66,67]. The methylation distributions of CpG are uneven in mammalian genomes. Although the methylation levels of the promoter regions were relatively low in this study, the methylation of promoter CpG islands (CGIs) often play vital roles in the expressions of related genes [68,69,70]. A study showed that the differences in the DEGs of human and chimpanzee are usually associated with differences in promoter methylation, and methylation differences in a small number of key genes may determine species-specific traits [71]. 

Numerous studies have focused on the link between dietary nutrition and DNA methylation in mammalians [72,73]. Due to practical limitations, we only compared methylation in the two pandas with ferret. From our current research results, the enrichment of the GO and KEGG analyses performed on the convergent promoters in the stomach and small intestine of the two pandas compared with ferret were mainly involved in the metabolic process, indicating that methylation may play a role in the digestion and metabolism of the nutrients in bamboo in the two pandas. SLC2A3, as a facilitative glucose transporter, may mediate the uptake of a variety of sugars such as glucose, galactose and xylose [74,75]. Carbohydrate-utilization-related *SLC2A3* in the stomach exhibits inverse mRNA expression and promoter methylation levels (Table S18 and Figure 8A,B), indicating that higher gene expressions of *SLC2A3* in the two pandas may be regulated by hypomethylated promoters to meet the energy demands that arise from eating low-lipid bamboo. The protease PARL may release the Lys or Arg residue from the carboxy terminal protein. *PARL* in the stomach exhibits inverse mRNA expression and promoter methylation levels (Table S18 and Figure 8C,D), indicating that higher gene expressions of *PARL* in the two pandas may be regulated by hypomethylated promoters to adapt to a bamboo diet with limited Lys and Arg content. Lipids emulsified into small fragments by bile are further digested into free fatty acids, glycerol, cholesterol and phospholipids by lipase, pancrelipase and cholesterol esterase in the small intestine [76,77]. After digestion, fatty acids and glycerol are absorbed via simple diffusion into the epithelial cells of the small intestine. Triglycerides, which are resynthesized by absorbed fatty acids and glycerol in the endoplasmic reticulum of the small intestine, form chylomicrons in the small intestine with cholesterol and phospholipids; then, they enter into the lymphatic circulation or are temporarily stored within cytoplasmic lipid droplets in enterocytes [51,78]. DGAT2 contributes to the resynthesis of triglyceride, the regulation of chylomicron assembly and the alteration of cytoplasmic lipid-droplet morphology in enterocytes, which may regulate dietary-fat absorption in the small intestine [79]. The down-regulated expression of *DGAT2*, related to lipid absorption, implies that both pandas are intolerant to a high-fat diet and adaptive to a low-lipid bamboo diet compared with non-herbivore species. Lipid-absorption-related *DGAT2* in the small intestine exhibits inverse mRNA expression and promoter methylation levels (Table S18 and Figure 8E,F), suggesting that lower gene expressions of *DGAT2* in the two pandas may be regulated by hypermethylated promoters to adapt to low-lipid bamboo.

Epigenetic information can be inherited through the mammalian germline, and the DNA methylation of offspring is influenced by the maternal diet, including changes in carbohydrates, lipids, amino acids and vitamins [80,81,82,83,84]. A long time ago, methylation modification may have regulated the expression of these genes in both pandas to adapt to the sudden environmental changes rapidly. Then, after a long period of natural selection and adaptive evolution, methylation modification in the two pandas may have been inherited by future generations to adapt to a long-term low-nutrition bamboo diet. 

## 5. Conclusions

In summary, our study provides new insights into the molecular mechanisms of the convergent evolution of giant panda and red panda, from their meat-eating ancestors to obligate bamboo-feeders, at the gene expression level via comparative transcriptomics of the stomach and small intestine among five species. We identified numerous convergent DEGs of the two pandas via RNA-seq associated with carbohydrate utilization, bile secretion, Lys and Arg metabolism, vitamin B12 utilization, and cyanide detoxification, which coincided with their adaptation to a low-nutrition and high-cyanide bamboo diet. Meanwhile, we analyzed the DNA methylation patterns in the stomach and small intestine of giant panda, red panda and ferret to investigate the changes in methylation on a genome-wide scale for the first time. We found that convergent promoter methylation, which possibly regulates the expressions of digestive and metabolic genes, may help shape specific feeding phenotypes and contribute to adaptive evolution in giant panda and red panda. 

## Data Availability

The RNA-seq and Bs-seq data of giant panda, red panda and ferret in this study have been submitted to the NCBI Sequence Read Archive (accession number PRJNA861855). The RNA-seq data of mouse and dog in this study are available from the NCBI Sequence Read Archive.

## Supplementary Materials

The following supporting information can be downloaded at: https://www.mdpi.com/article/10.3390/genes13081446/s1, Figure S1: Distributions of coefficient of variation in gene expression levels in stomach and small intestine samples before and after normalization of all orthologous genes; Figure S2: Volcano plots of DEGs in stomach (A) and small intestine (B); Figure S3: Accumulated distribution of sequence coverage in stomach (A) and small intestine (B) in genomes of giant panda, red panda and ferret; Figure S4: Summary of CG, CHG and CHH contexts of giant panda, red panda and ferret; Figure S5: PCA and clustering analyses of the promoter methylation levels for giant panda, red panda and ferret; Figure S6: Promoter methylation patterns in stomach and small intestine samples; Table S1: Description of the 32 RNA-seq libraries for five mammals used in this study; Table S2: Statistics of orthologous gene numbers detected using Orthofinder software; Table S3: Sequence coverage of orthologous genes among five species; Table S4: Identity of orthologous genes among five species; Table S5: Normalized expression levels of 306 convergent DEGs in stomach samples of two panda species; Table S6: Normalized expression levels of 150 convergent DEGs in small intestine samples of two panda species; Table S7: Significantly enriched GO categories for up-regulated convergent DEGs in stomach samples of two panda species; Table S8: Significantly enriched GO categories for down-regulated convergent DEGs in stomach samples of two panda species; Table S9: Significantly enriched GO categories for down-regulated convergent DEGs in small intestine samples of two panda species; Table S10: Significantly enriched KEGG pathways for down-regulated convergent DEGs in small intestine samples of two panda species; Table S11: Description of the 20 BS-seq libraries for three mammals used in this study; Table S12: The proportion of C sites in the genomes of giant panda, red panda and ferret covered by at least 1 and 5 unique reads; Table S13: Significantly enriched GO categories for hypomethylated convergent promoters in stomach samples of two panda species; Table S14: Significantly enriched GO categories for hypermethylated convergent promoters in stomach samples of two panda species; Table S15: Significantly enriched KEGG pathways for hypermethylated convergent promoters in stomach samples of two panda species; Table S16: Significantly enriched GO categories for hypomethylated convergent promoters in small intestine samples of two panda species; Table S17: Significantly enriched GO categories for hypermethylated convergent promoters in small intestine samples of two panda species; Table S18: DEGs associated with carbohydrate utilization, bile secretion, lipid absorption, Arg and Lys metabolism, vitamin B12 utilization and cyanide detoxification in stomach and small intestine.
