# Supplementary material for: Comparative Transcriptomics and Methylomics Reveal Adaptive Responses of Digestive and Metabolic Genes to Dietary Shift in Giant and Red Pandas
Source: Genes (Basel). 2022 Aug 14;13(8):1446. doi: 10.3390/genes13081446 (PMC9407821; doi:10.3390/genes13081446)
Supplement: Supplementary file 1 [file genes-13-01446-s001.zip › Supplementary figures.pdf]

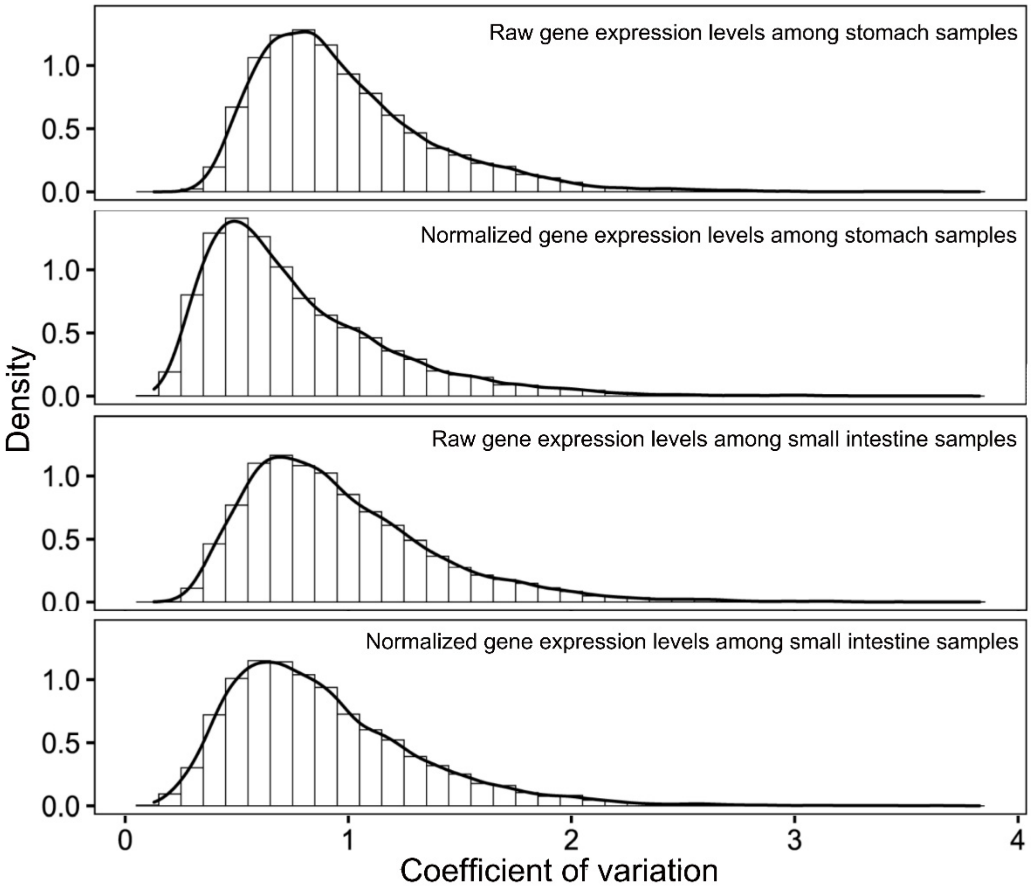

Figure S1. Distributions of coefficient of variation in gene expression levels in stomach and small intestine samples before and after normalization of all orthologous genes. A normal density curve was based on the fitting result of the histogram.

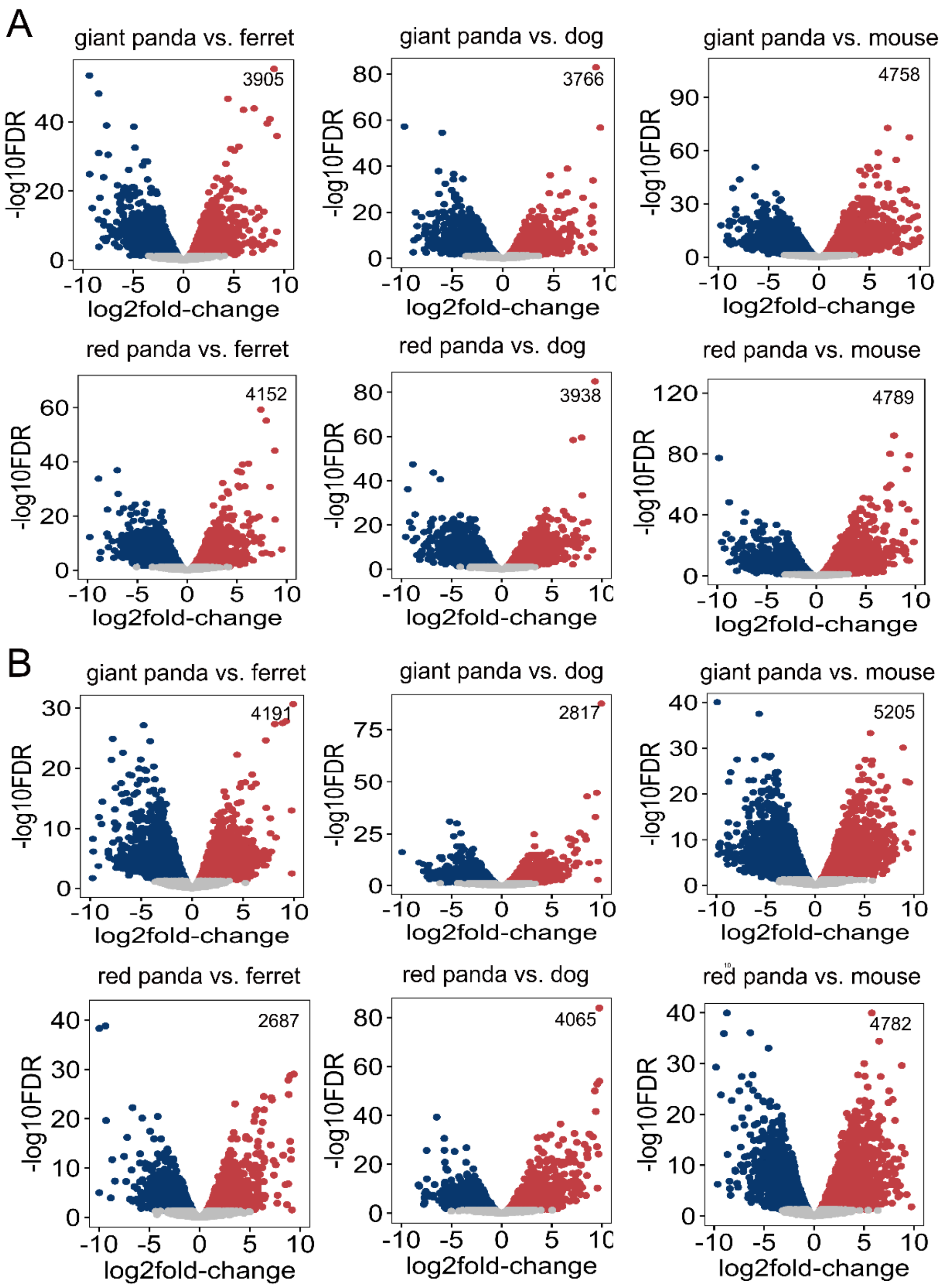

Figure S2. Volcano plots of DEGs in stomach (A) and small intestine (B). Each dot represents one gene. Red dots represent up-regulated DEGs, blue dots represent down-regulated DEGs, and grey dots represent no significantly biased genes. All numbers in the upper right corner indicate the number of DEGs in the pairwise comparison.

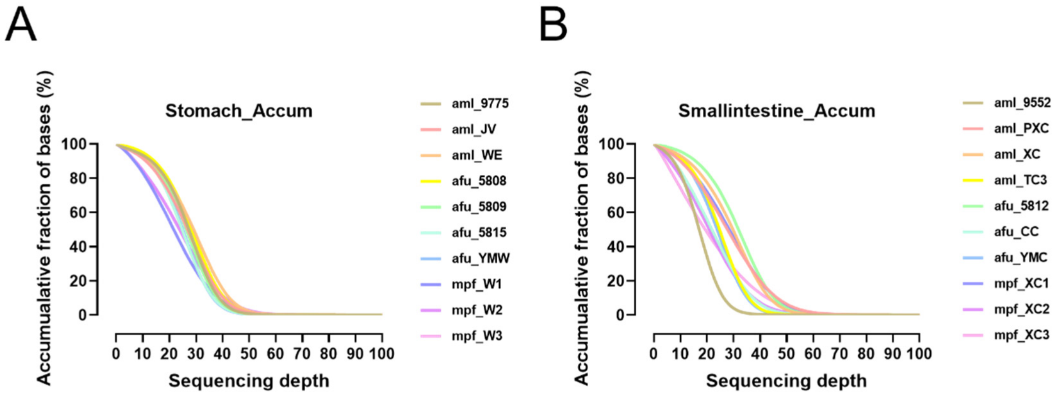

Figure S3. Accumulated distribution of sequence coverage in stomach (A) and small intestine (B) in genomes of giant panda, red panda and ferret. The abscissa represents the coverage depth and the ordinate represents the proportion of the coverage not less than the depth of the total number of base sites.

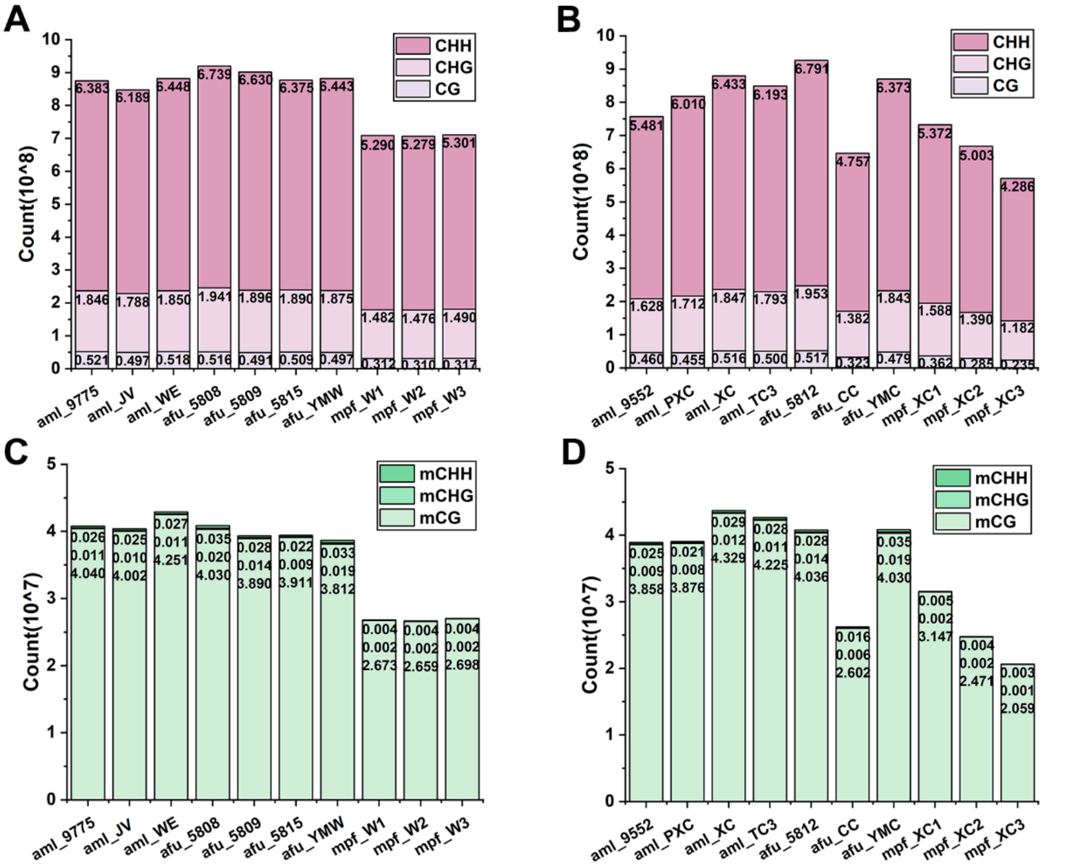

Figure S4. Summary of CG, CHG and CHH contexts of giant panda, red panda and ferret. The bar chart shows the number of cytosines in CG, CHG and CHH sites covered by at least 5 reads per sample from stomach (A). The bar chart shows the number of cytosines in CG, CHG and CHH sites covered by at least 5 reads per sample from small intestine (B). The bar chart shows the number of CG, CHG and CHH sites with methylation levels greater than 50% covered by at least 5 reads per sample from stomach (C). The bar chart shows the number of CG, CHG and CHH sites with methylation levels greater than 50% covered by at least 5 reads per sample from small intestine (D).

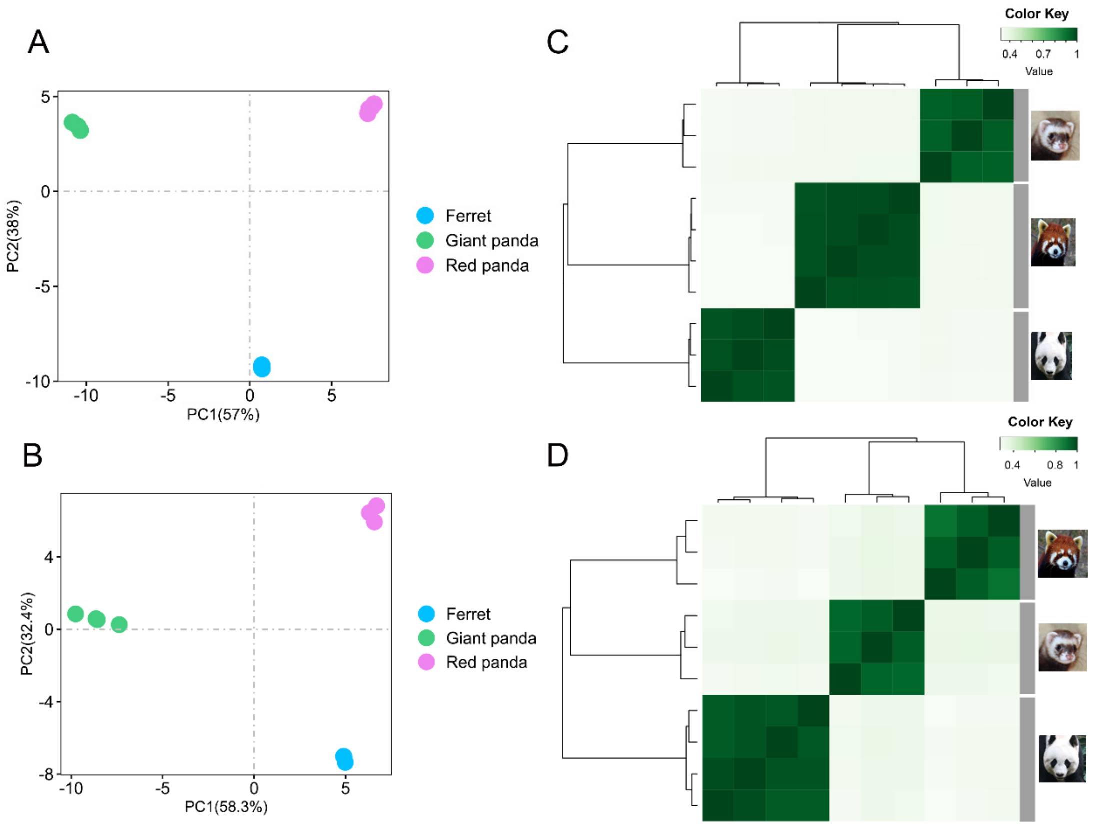

Figure S5. PCA and clustering analyses of the promoter methylation levels for giant panda, red panda and ferret.

The promoter methylation data of all orthologous genes were used to perform PCA analyses in stomach (A) and small intestine (B) samples of giant panda, red panda and ferret. Different species are represented by circles of different colors. The promoter methylation data of all orthologous genes were used to perform clustering analyses in stomach (C) and small intestine (D) samples of giant panda, red panda and ferret. Distance between samples was measured by Spearman's rank correlation coefficient.

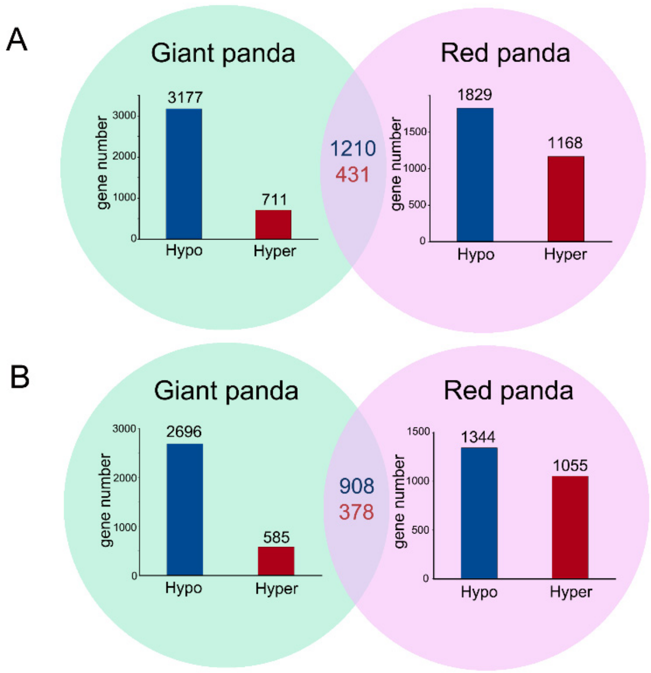

Figure S6. Promoter methylation patterns in stomach and small intestine samples. Bar plots in green and purple circles indicate the numbers of differentially methylated promoters of giant panda and red panda compared with ferret in stomach samples, respectively. Numbers in red and blue indicate hypermethylated and hypomethylated convergent promoters of two pandas compared with ferret in stomach samples, respectively (A). Bar plots in green and purple circles indicate the numbers of differentially methylated promoters of giant panda and red panda compared with ferret in small intestine samples, respectively. Numbers in red and blue indicate hypermethylated and hypomethylated convergent promoters of two pandas compared with ferret in small intestine samples, respectively (B).
